# Advancing Endodormancy Release in Temperate Fruit Trees Using Agrochemical Treatments

**DOI:** 10.3389/fpls.2021.812621

**Published:** 2022-01-14

**Authors:** Jesús Guillamón Guillamón, Federico Dicenta, Raquel Sánchez-Pérez

**Affiliations:** Department of Plant Breeding, CEBAS-CSIC, Campus Universitario de Espinardo, Espinardo, Spain

**Keywords:** agrochemical, chill, endodormancy, hydrogen cyanamide, temperate fruit trees

## Abstract

Endodormancy in temperate fruit trees like *Prunus* is a protector state that allows the trees to survive in the adverse conditions of autumn and winter. During this process, plants accumulate chill hours. Flower buds require a certain number of chill hours to release from endodormancy, known as chilling requirements. This step is crucial for proper flowering and fruit set, since incomplete fulfillment of the chilling requirements produces asynchronous flowering, resulting in low quality flowers, and fruits. In recent decades, global warming has endangered this chill accumulation. Because of this fact, many agrochemicals have been used to promote endodormancy release. One of the first and most efficient agrochemicals used for this purpose was hydrogen cyanamide. The application of this agrochemical has been found to advance endodormancy release and synchronize flowering time, compressing the flowering period and increasing production in many species, including apple, grapevine, kiwi, and peach. However, some studies have pointed to the toxicity of this agrochemical. Therefore, other non-toxic agrochemicals have been used in recent years. Among them, Erger^®^ + Activ Erger^®^ and Syncron^®^ + NitroActive^®^ have been the most popular alternatives. These two treatments have been shown to efficiently advance endodormancy release in most of the species in which they have been applied. In addition, other less popular agrochemicals have also been applied, but their efficiency is still unclear. In recent years, several studies have focused on the biochemical and genetic variation produced by these treatments, and significant variations have been observed in reactive oxygen species, abscisic acid (ABA), and gibberellin (GA) levels and in the genes responsible for their biosynthesis. Given the importance of this topic, future studies should focus on the discovery and development of new environmentally friendly agrochemicals for improving the modulation of endodormancy release and look more deeply into the effects of these treatments in plants.

## Introduction

**FIGURE 1:**
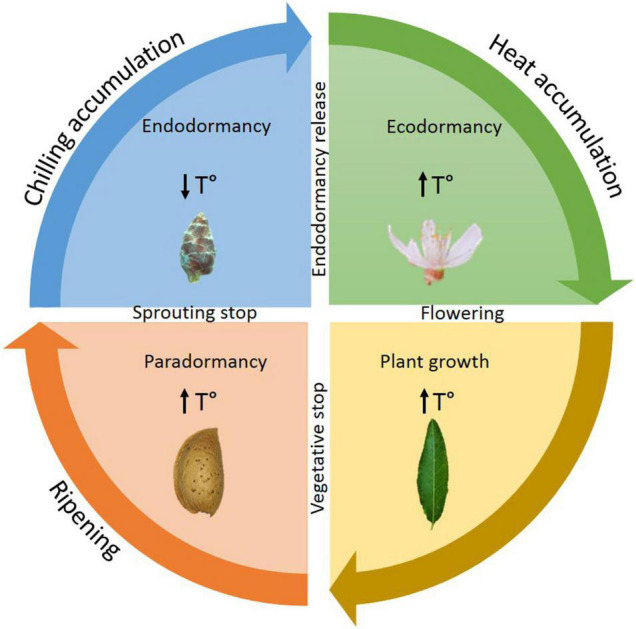
Dormancy cycle and plant growth. Events that take place in the different stages of dormancy cycle and plant growth.

Endodormancy in perennial trees, like *Prunus*, is an essential step for plant survival in the unfavorable conditions of autumn and winter (Beauvieux et al., 2018). The dormancy cycle is divided into three different stages: endodormancy, ecodormancy, and paradormancy (Lang et al., 1987; Fadón et al., 2020; Figure 1). Endodormancy is controlled by the bud itself and is characterized by slow metabolism and suspended growth in roots, stems, leaves, and flowers (Martínez-Gómez et al., 2017). Throughout this stage, chill is gathered by the plant; the flower and vegetative buds will only be able to release from endodormancy once the chilling requirements (CR) have been fulfilled (Prudencio et al., 2018). The CR are characteristic of each cultivar, and incomplete fulfillment might provoke imperfections in flowering and production, which translate into great losses for farmers (Fennell, 1999). Once endodormancy has released, the resumption of growth is still inhibited by unfavorable external conditions, rather than internal cues. Once the adverse environmental conditions of winter end, the warm temperatures of spring trigger the development of the future flowers as well as tree growth. This stage between endodormancy release and flowering is known as ecodormancy (Szalay et al., 2010; Liu and Sherif, 2019). The last stage of the cycle is paradormancy, which takes place throughout summer and ends in the final summer weeks. In this stage, lateral growth is inhibited by apical dominance as well as auxins (Champagnat, 1983).

Temperature and photoperiod are probably the main cues that trigger the stages of the dormancy cycle, including endodormancy release and flowering (Walsh et al., 2002; Liu and Sherif, 2019). As a result, global warming has placed a huge amount of pressure on perennial trees. Unusually high temperatures during winter do not provide the necessary chill requirements, causing imperfect, asynchronous and deficient flowering, which results in a drop in production and fruit quality (Blanke and Kunz, 2009; Martínez-Gómez et al., 2017).

Thus, with the aim of neutralizing the effects of climate change, breeding programs are releasing low-CR cultivars (early flowering) in species like peach (*Prunus persica* L. Batsch), sweet cherry [*Prunus avium* (L.) L.], plum (*Prunus domestica* L.), and apricot (*Prunus armeniaca* L.). The new genotypes will be able to fulfill their CR even under increased temperatures caused by global warming (Martínez-Calvo et al., 2009). Nevertheless, breeding programs are costly and time-consuming—it takes an average of 10–15 years to register a new cultivar (Dicenta et al., 2009).

Different products are therefore applied to crops to promote endodormancy release (Rodrigo et al., 2017). These products—called agrochemicals, plant growth regulators or biostimulants—are organic compounds used for advancing endodormancy release and flowering, synchronizing, and compressing the latter. They usually consist of high concentrations of nitrogen from distinct sources (Table 1). However, other agrochemicals with different bases have been tested, obtaining different results in terms of endodormancy release, and flowering advance (Hopping, 1977; Erez et al., 2008; Botelho et al., 2016; Bound and Miller, 2016).

**TABLE 1:** Composition of the different agrochemicals used for advancing endodormancy release and species in which they have been tested.

Agrochemical	Composition	Species	Concentration applied (%)	Volume per ha	References
Dormex^®^	52% Hydrogen cyanamide	Apple, grapevine, blackberry, sweet cherry, almond, and peach	0.5–5	650–1000 L ha^–1^	Jackson and Bepete, 1995; Segantini et al., 2015; Pasa et al., 2018; Shi et al., 2018
Erger^®^	3.1% Ammoniacal nitrogen + 5.8% Nitrate nitrogen + 6.1% Urea nitrogen + 3.3% Calcium	Apple, grapevine, kiwi, blackberry, sweet cherry, and peach	2–7	650–1000 L ha^–1^	Erez et al., 2008; Segantini et al., 2015; Ardiles and Ayala, 2017; Ferreira et al., 2019; Fowler et al., 2020
Activ Erger^®^	9% Nitric nitrogen + 6% Ammoniacal nitrogen + 6.5% CaO	Apple, grapevine, kiwi, blackberry, sweet cherry, and peach	6–8	650–1000 L ha^–1^	Erez et al., 2008; Segantini et al., 2015; Ardiles and Ayala, 2017; Ferreira et al., 2019; Fowler et al., 2020
Syncron^®^	2% Free aminoacids + 0.3% Total nitrogen + 80% Total organic matter	Apple and sweet cherry	1–2	1000 L ha^–1^	Petri et al., 2016; Ardiles and Ayala, 2017
NitroActive^®^	11.5% Nitric nitrogen + 5.5% Ammoniacal nitrogen + 12.3% CaO	Apple and sweet cherry	3–20	1000 L ha^–1^	Petri et al., 2016; Ardiles and Ayala, 2017
Bluprins^®^	0.7% Free aminoacids + 4% Nitric nitrogen + 4% Ammoniacal nitrogen + 5.5% Organic carbon	Kiwi	Not specified	30 L ha^–1^	Tavares et al., 2018
Bluact^®^	9% Nitric nitrogen + 6% Ammoniacal nitrogen + 7% CaO	Kiwi	Not specified	120 L ha^–1^	Tavares et al., 2018
Kiplant HB15^®^	9% Nitric nitrogen + 6% Ammoniacal nitrogen + 6.1% CaO	Kiwi	Not specified	24 L ha^–1^	Tavares et al., 2018
Kiplant Inducer^®^	6.5% Nitric nitrogen + 3% Ammoniacal nitrogen + 6.1% Urea nitrogen + 6.1% CaO	Kiwi	Not specified	36 L ha^–1^	Tavares et al., 2018
Siberio^®^	6% Nitric nitrogen + 3% Ammoniacal nitrogen + 6% Urea nitrogen + 4.5% CaO + 0.05% Fe + 0.05% Zn	Kiwi	Not specified	40 L ha^–1^	Tavares et al., 2018
Siberion^®^	9% Nitric nitrogen + 6% Ammoniacal nitrogen + 7% CaO	Kiwi	Not specified	90 L ha^–1^	Tavares et al., 2018
W-Uniformity Superplus^®^	2.3% Free aminoacids + 7.6% Nitric nitrogen + 2.4% Ammoniacal nitrogen + 2.6% Urea nitrogen + 8% CaO + 0.02% Fe + 0.002% Zn + 2.8% K_2_O + 0.015% Mn + 0.07% MgO	Kiwi	Not specified	20–30 L ha^–1^	Tavares et al., 2018
Organihum Plus^®^	Total nitrogen 1.5% + 4.4% Organic carbon + 6% K_2_O + 6% P_2_O_5_ + 0.7% B	Kiwi	Not specified	0.5 L ha^–1^	Tavares et al., 2018
Organihum B-Plus^®^	17.5% B	Kiwi	Not specified	1.5 Kg ha^–1^	Tavares et al., 2018
Sitofex^®^	0.12% Forchlorfenuron	None	None	None	None
Waiken™	38.8% Methyl esters of fatty acids	Apple and sweet cherry	4	1000–2000 L ha^–1^	Bound and Miller, 2006, 2016
Thiourea	Thiourea	Grapevine and peach	1–5	0.25 g ha^–1^	Hopping, 1977
DROPP^®^	42.4% Thidiazuron	Sweet cherry and peach	0.04	0.12–0.16 L ha^–1^	Petri et al., 2014
Natur’oleo^®^	93% Vegetable oil	Grapevine	2	0.3–1.5 L ha^–1^	Botelho et al., 2016
Mineral oil	83% Parafinic oil	Apple, grapevine, blackberry, sweet cherry, almond, and peach	2–4	650–1000 L ha^–1^	Erez and Zur, 1981; Küden et al., 1995; Sagredo et al., 2005; Petri et al., 2014; Botelho et al., 2016; Hernández and Craig, 2016
Armobreak™	22% Alkylamine polymer + 23% organic nitrogen + 40% inorganic nitrogen	Kiwi, sweet cherry, peach, and pistachio	2	650–1000 L ha^–1^	Ionescu et al., 2017a; López Alcolea, 2018

## Effects of Agrochemicals on Endodormancy Release

In recent years, many agrochemical treatments have been performed in warm areas in order to shorten endodormancy and advance flowering time (Ardiles and Ayala, 2017; Rodrigo et al., 2017; Ferreira et al., 2019). This practice presents significant problems, however, owing to a lack of knowledge about the efficiency of the treatments and the optimal concentrations and application times for distinct species and cultivars (Ionescu et al., 2017a; Rodrigo et al., 2017; Pasa et al., 2018). In order to assist the promotion of endodormancy release in these treatments, other agrochemicals like Armobreak^®^ or mineral oil have been used to prevent evaporation and help ensure that the treatments penetrate the plant (Küden et al., 1995; Sagredo et al., 2005; López Alcolea, 2018). Several works in kiwi, pistachio (*Pistacia vera* L.), apple and *Prunus* spp. have indicated that the optimal concentration of agrochemicals like Armobreak™ and mineral oil is 3% (Erez and Zur, 1981; Küden et al., 1995; Sagredo et al., 2005; Erez et al., 2008; Petri et al., 2014; Botelho et al., 2016; Hernández and Craig, 2016). Nevertheless, these products have not solved the problems linked to the promotion of endodormancy release. Recent works have therefore focused on the effects and the application parameters (application time, concentration, species, phytotoxicity, and so on) of these agrochemicals in many crops (Hawerroth et al., 2010; Rademacher, 2015; Segantini et al., 2015; Petri et al., 2016; Pasa et al., 2018).

### Hydrogen Cyanamide: The First Agrochemical Used to Promote Endodormancy Release

Hydrogen cyanamide, mostly sold as Dormex^®^ (520 g/l hydrogen cyanamide, AlzChem, Germany) (Table 1), has been widely used for assisting chill accumulation and synchronizing flowering time in many species, including apple (*Malus domestica* Borkh.), grapevine (*Vitis vinifera* L.), blackberry (*Rubus* spp.), sweet cherry, almond, and peach (Jackson and Bepete, 1995; Segantini et al., 2015; Ionescu et al., 2017a; Shi et al., 2018).

In apple, endodormancy release and flowering time were significantly earlier than in control trees when different hydrogen cyanamide solutions (1.5–4%) were applied (Jackson and Bepete, 1995). These differences were even more pronounced in cultivars with high CR, in which flowering occurred more than 7 days earlier in treated trees than in control trees (Jackson and Bepete, 1995; Đmrak et al., 2016). Under warm winter conditions, the differences in flowering time were even greater (Đmrak et al., 2016). In other species like grapevine, buds treated with a hydrogen cyanamide solution (5%) exhibited a higher flower bud break ratio than the control buds (Ophir et al., 2009). Other crops, such as peach, have displayed the same behavior as apple and grapevine after treatment with a hydrogen cyanamide solution (1–2%). In these studies, the flower bud break ratio was between 27 and 57% higher in treated than in control trees (Dozier et al., 1990; Yooyongwech et al., 2012).

However, despite all of these promising results, it must be said that hydrogen cyanamide is poisonous for humans—it can produce a disulfiram-like syndrome, in which acetaldehyde accumulates in the blood, producing headaches, low blood pressure, palpitations, nausea, and chest pain (Mergenhagen et al., 2020). Its use has therefore been banned in many countries worldwide (Sheshadri et al., 2011), including European Union countries, New Zealand, and more.

### Alternatives to Hydrogen Cyanamide Based on Nitrogen Compounds

Due to the toxicity of hydrogen cyanamide, numerous agrochemicals for promoting endodormancy release have been tested and brought out (Sheshadri et al., 2011; Ardiles and Ayala, 2017; Rodrigo et al., 2017). Most of these agrochemicals contain high concentrations of nitrogen from nitrite salts and amino acids (Table 1). Among these agrochemicals, the most popular are Erger^®^ + Activ Erger^®^ and Syncron^®^ + NitroActive^®^ (Rodrigo et al., 2017; Ferreira et al., 2019). However, new agrochemicals with similar formulas have also been used to a lesser extent, such as Bluprins^®^ + Bluact^®^, Kiplant HB15^®^ + Kiplant Inducer^®^, Siberio^®^ + Siberion^®^ and W-Uniformity Superplus^®^ (Tavares et al., 2018; Table 1).

Trees treated with these compounds suffer from aggressive oxidative stress in bud cells, which results in metabolic rearrangements. At the same time, the ATP anabolism is increased by glycolysis and fermentation, promoting endodormancy release (Pérez et al., 2009; Segantini et al., 2015).

Erger^®^ + Activ Erger^®^ have been widely applied in many different species like apple, grapevine, kiwi [*Actinidia deliciosa* (A.Chev.) C.F.Liang & A.R.Ferguson], blackberry, sweet cherry and peach to promote endodormancy release, and flowering (Segantini et al., 2015; Ardiles and Ayala, 2017; Pasa et al., 2018; Ferreira et al., 2019; Fowler et al., 2020).

In apple, treatment with Erger^®^ + Activ Erger^®^ (3–7%) and Syncron^®^ + NitroActive^®^ (1–2%) has been found to advance endodormancy release and flowering in several cultivars under cold and warm winter conditions (Hawerroth et al., 2010; Petri et al., 2016; Đmrak et al., 2016; Abreu et al., 2018) compared to positive control trees treated with a hydrogen cyanamide solution. In cold winters, the Erger^®^ + Activ Erger^®^ combination was more efficient than hydrogen cyanamide, whereas in warm winters, no differences were found between the treatments. Nevertheless, even in warm winters, both treatments produced an earlier endodormancy release than the untreated control (Hawerroth et al., 2010).

Also in apple, Syncron^®^ + NitroActive^®^ produced the same effects as hydrogen cyanamide, independently of the weather conditions (Petri et al., 2016). On the contrary, a 3-year study with warm and cold years showed an earlier full bloom in control trees than in trees treated with either Erger^®^ (2–6%) or hydrogen cyanamide (0.34%) solutions (Pasa et al., 2018). However, it must be said that trees treated with the highest Erger^®^ concentrations showed higher production levels than the control trees, as previously shown (Đmrak et al., 2016).

In agreement with these results, it has been observed in grapevine that, under subtropical conditions, plants sprayed with an Erger^®^ solution, and plants treated with hydrogen cyanamide (3.5–4.5%) solutions present similar vegetative bud break rates (Fowler et al., 2020). Even so, both Erger^®^ and hydrogen cyanamide treatments exhibited a higher budburst (10–15%) than the control treatment (Fowler et al., 2020; Rosa et al., 2020).

The same results were observed in blackberry when plants were treated with hydrogen cyanamide (2–8%) or Erger^®^ (2–8%) solutions. All plants treated with either of these solutions had an earlier endodormancy release and flowering and harvest time than control trees (Segantini et al., 2015). These findings support the viability of Erger^®^ as an alternative endodormancy release promotor. Nevertheless, they still need to be validated in further studies, using more species and cultivars in years with different levels of chill accumulation.

Erger^®^ has also been used to promote endodormancy release in kiwi. Under cold winter conditions, kiwi trees treated with an Erger^®^ (6%) solution presented a higher flower bud break rate than those treated with a hydrogen cyanamide solution (5%) (Hernández and Craig, 2016). In contrast to Erger^®^, the Syncron^®^ + NitroActive^®^ treatment has not yet been found to produce an earlier endodormancy release in this species. This might be due to deficient chill accumulation, since less than 40% of the CR were fulfilled in this study (Tavares et al., 2018).

Other less popular agrochemicals have also been used to advance endodormancy release, including Bluprins^®^ (Biolchim, Italy) + Bluact^®^ (Biolchim, Italy), Kiplant HB15^®^ (Asfertglobal, Portugal) + Kiplant Inducer^®^ (Asfertglobal, Portugal), Siberio^®^ (Green Hass, Italy) + Siberion^®^ (Green Has, Italy), and W-Uniformity Superplus^®^ (Agroserna S.L., Spain) (Table 1). To date, kiwi is the only species in which these treatments have been applied (Tavares et al., 2018). The first two treatments produced a significant increase in flower bud break percentage compared to the control trees, whereas the rest of the treatments did not present any differences. Nevertheless, in all of the treatments, the sprayed trees showed lower production levels than the control trees (Tavares et al., 2018).

As in other species, the use of Erger^®^ (6%) or Syncron^®^ (2%) + NitroActive^®^ (20%) has been found to advance endodormancy release in sweet cherry compared to control trees (Ardiles and Ayala, 2017; Rodrigo et al., 2017). Moreover, when trees were treated with an Erger (6%) solution, they flowered 1 week earlier than trees treated with a hydrogen cyanamide solution (2%).

### Agrochemicals With Alternative Chemical Bases

In recent years, several new agrochemicals have been released based on either carbon, potassium and boron [Organihum Plus^®^ (Econatur, Spain) + Organihum B-Plus^®^ (Econatur, Spain)]; nitrogen and sulfur (thiourea); vegetable oils [Waiken™ (SST Australia Pty Ltd, Australia) and Natur’oleo^®^ (Stoller, United States)]; or more complex compounds like cytokinins [DROPP^®^ (Bayer, Germany) and Sitofex^®^ (BASF, Spain)] (Table 1). Waiken™ is an emulsion of methyl esters of fatty acids that has been tested in species like apple and sweet cherry (Bound and Miller, 2006, 2016). The principal effect of this agrochemical when applied in early endodormancy is homogenous flowering. However, results in terms of promoting endodormancy release and flowering have varied greatly from year to year, and advances in both only occurred in the years the treatment was applied in the early stages of endodormancy (Bound and Miller, 2006). Thioureas are compounds with high concentrations of nitrogen and sulfur that produce some advances in endodormancy release in grapevine. This effect is even greater when high concentrations of thioureas are used (Hopping, 1977). Another two agrochemicals, DROPP^®^ and Sitofex^®^, are phenylurea-derivatives, mixed with different cytokinins like thidiazuron and forchlorfenuron, respectively. DROPP^®^ has been useful in advancing endodormancy release and flowering in some *Prunus* species (Erez et al., 2008). However, no studies in flower buds have been published to date using Sitofex^®^ as an endodormancy release promoter.

## Effects of the Different Agrochemicals on Production

Many studies performed in different species have revealed that these agrochemicals mentioned above have a great impact on fruit tree production (Đmrak et al., 2016; Tavares et al., 2018). In apple, high concentrations of hydrogen cyanamide or Erger^®^ significantly increased production, resulting in a greater number of fruits with a similar weight than in control trees (Jackson and Bepete, 1995; Seif El-Yazal and Rady, 2012; Đmrak et al., 2016). In kiwi, hydrogen cyanamide and Erger^®^ also boosted production, resulting in bigger fruits and also a more uniform fruit size than in control trees (Ardiles and Ayala, 2017). Furthermore, alternative treatments in grapevine using the vegetable oil Natur’oleo^®^ + mineral oil or thioureas have been found to increase the annual yield compared to untreated plants (Botelho et al., 2016). In contrast to these results, kiwi trees treated with Syncron^®^ + NitroActive^®^ and the less popular agrochemicals that promote endodormancy release (Bluprins^®^ + Bluact^®^, Kiplant HB15^®^ + Kiplant Inducer^®^, Siberio^®^ + Siberion^®^, W-Uniformity Superplus^®^, and Organihum Plus^®^ + Organihum B-Plus^®^) showed a decrease in production compared to the control trees (Tavares et al., 2018).

In *Prunus* spp., several studies have confirmed that hydrogen cyanamide can also increase fruit production and quality. For instance, a study in peach showed that the application of a hydrogen cyanamide solution (3%) had a great impact on fruit quality, increasing the fruit set and the fruit weight by over 30%, and decreasing fruit drop by 50% (Singh and Banyal, 2020). Similar results have also been observed in apricot and plum, in which the application of a hydrogen cyanamide solution (3%) increased the fruit set of the treated trees in both species (Son and Kuden, 2005; Kelany et al., 2009).

Furthermore, sweet cherry trees were studied in two different locations under warm and cold winter conditions. In warm winter areas, some trees were treated with a 2% hydrogen cyanamide solution to test its effects on flower organ development. Organ damage was mostly observed in the ovule and embryo sac of the control trees, and 84% of flowers were not sufficiently developed for fertilization versus 48% on the treated trees. In cold winter areas, the percentage of undeveloped flowers was around 50% on treated and control trees. These results confirm the usefulness of hydrogen cyanamide for correcting flower development in warm winter areas, where chill accumulation is not sufficient to ensure proper flowering and production (Wang et al., 2016).

## Agrochemical Application Time and Concentration

A study performed using endodormancy release promoters indicated two crucial factors for successful treatment: the application time and the concentration used (Jackson and Bepete, 1995).

Regarding the application time, a 6% Erger^®^ solution was applied in kiwi at three different time points. Under cold winter conditions, the most effective application time was 30 days before flower bud break. On the contrary, under warm winter conditions, the most effective application time was 61 days before flower bud break (Hoeberichts et al., 2017). This dissimilarity might be due to the distinct levels of accumulated chill in each winter, since an incomplete fulfillment of CR produces abnormal bud break, flowering, and production (Fennell, 1999).

These results agree with a work in sweet cherry, in which trees from two cultivars were treated with a 2% Syncron^®^ + 20% NitroActive^®^ solution at two application times for 2 years. In the cultivar with the latest flowering time, the later application in the endodormancy period produced a greater advance in flowering than the earlier one under both cold and warm winter conditions, whereas it was only effective in the earliest flowering cultivar under warm winter conditions (Rodrigo et al., 2017). As in the case of kiwi, this difference may be due to the different levels of chill accumulated in each winter. Under cold winter conditions, the CR of the earliest cultivar might have already been fulfilled when the agrochemical was applied, thus producing no variation in flowering time.

In addition, there is significant controversy about the optimal application date. Several studies in peach and apple have indicated the necessity of a certain amount of chill accumulation prior to agrochemical application (Siller-Cepeda et al., 1992; Bound and Jones, 2004). However, the best way to measure this chill accumulation prior to application remains unclear. Some works have suggested applying the treatment at a certain number of weeks before the average endodormancy release date (Bound and Jones, 2004). In our opinion, this is not a valid way to determine the application time, since the chill accumulation during these weeks is totally dependent on the year and location. On the other hand, recent studies in grapevine, sweet cherry, and apricot have stated that the treatment should be applied when two-thirds of the CR have been fulfilled (Ben Mohamed et al., 2012; Ionescu et al., 2017a,b; López Alcolea, 2018). Nevertheless, this still needs to be validated in more species and cultivars, since huge differences in CR may affect the optimal application time.

The concentration, as we mentioned before, is the second crucial factor in successfully advancing endodormancy release. In apricot, a comparison of 1, 2, and 3% hydrogen cyanamide solutions showed that 3% produced the highest fruit set (Kelany et al., 2009). This fact agrees with other results in peach and plum, in which trees treated with a 2% solution exhibited a higher flower bud break rate than trees treated with a 1% solution (Dozier et al., 1990; Son and Kuden, 2005).

This trend was also observed in other assays using Erger^®^ instead of hydrogen cyanamide (Hawerroth et al., 2010). Different works in apple demonstrated that high concentrations of Erger^®^ (5, 6, and 7%) were more efficient in advancing flowering time and increasing production (Hawerroth et al., 2010; Pasa et al., 2018). On the other hand, studies in grapevine and blackberry found no differences between low and high concentrations of Erger^®^ solutions in terms of flowering time, fruit ripening or production (Segantini et al., 2015; Rosa et al., 2020). Similarly, in apple, no dissimilarities were observed between trees treated with 1 and 2% Syncron^®^ solutions (Petri et al., 2016). Nevertheless, this lack of differences might be due to the low concentration applied, given that the optimal concentration of other agrochemicals with a similar composition is around 6% for apple, for example (Hawerroth et al., 2010; Pasa et al., 2018). All of these results confirm that stone fruits and apple trees need a high concentration of agrochemicals to obtain maximum performance (Dozier et al., 1990; Son and Kuden, 2005; Kelany et al., 2009; Hawerroth et al., 2010; Pasa et al., 2018). This is indeed problematic as it has been demonstrated that treatments with a high agrochemical concentration or an incorrect application time may cause bud toxicity, producing bud fall and huge production losses (Wang et al., 2016). Works in apple and peach have proven that high concentrations or late applications of aggressive agrochemicals like hydrogen cyanamide produce phytotoxicity, decreasing the total yield of that year due to destruction of the flower buds (Shulman et al., 1986; Dozier et al., 1990; Siller-Cepeda et al., 1992; Finetto, 1993; Fallahi et al., 1997; Bound and Jones, 2004). Therefore, these agrochemicals should always be applied according to the manufacturer’s indications.

## Biochemical and Genetic Variations After Agrochemical Application

In recent years, several studies have focused on the biochemical and genetic variations produced by the application of agrochemicals for promoting endodormancy release (Pérez et al., 2008; Kaufmann and Blanke, 2017). In apple, the application of a hydrogen cyanamide (4%) solution produced a significant increase in soluble nitrogen and polyamines in the flower buds (Seif El-Yazal and Rady, 2012). These results were—to some extent—expected, since hydrogen cyanamide is a nitrogen compound, which increases oxidative stress in plants (Segantini et al., 2015; Đmrak et al., 2016).

Studies in grapevine have revealed that plants treated with hydrogen cyanamide suffer changes in reactive oxygen species (ROS) levels and in the expression of some genes like α-amylase genes (Pérez et al., 2008; Vergara et al., 2012; Yooyongwech et al., 2012; Rubio et al., 2014; Zheng et al., 2015; Sudawan et al., 2016). Furthermore, the up- and downregulation of genes related to hypoxia and the biosynthesis of ABA and GA was detected in treated plants (Ophir et al., 2009). These results agree with other studies in almond and grapevine, which found that ABA, ROS, and GA play a crucial role in the endodormancy release process (Pérez et al., 2008; Guillamón et al., 2020). Overall, these results confirm that hydrogen cyanamide simulates hypoxia conditions in treated plants, increasing the expression of different groups of genes, such as hypoxic responsive genes, genes involved in the GA biosynthesis, and α-amylase genes, among others (Ophir et al., 2009; Vergara et al., 2012; Rubio et al., 2014).

Another study in peach showed that hydrogen cyanamide may enhance an influx of water to the dormant buds, causing the accumulation of water in the basal part of the peach flower (Yooyongwech et al., 2012). In sweet cherry, an accumulation of water during endodormancy release has been observed. Such water storage in buds right before, during and after endodormancy release could be due to a high concentration of carbohydrates, which would act as an osmotic factor in flower buds, producing water accumulation (Kaufmann and Blanke, 2017).

A recent study in sweet cherry focused on the metabolite and transcriptome variations that occur in flower buds after treatment with hydrogen cyanamide (Ionescu et al., 2017a). In this work, the metabolites from flower buds were extracted and analyzed by LC-MS. The results of these analyses pointed to prunasin and the phytohormone jasmonic acid as two principal actors in endodormancy release due to their significant variation during this process (Ionescu et al., 2017a). Prunasin variation was also observed in almond throughout the transition from endodormancy to ecodormancy (Del Cueto et al., 2017; Guillamón et al., 2020).

Regarding Erger^®^, only one study has investigated the differential expression of genes after its application (Hoeberichts et al., 2017). In this research, kiwi flower buds were analyzed by RNAseq. The results showed that genes responsible for seed germination, drought, and biotic stress exhibited significant downregulation. This fact agrees with other studies in grapevine and rice in which genes involved in the biosynthesis of ABA were downregulated during endodormancy and seed dormancy release, respectively (Ophir et al., 2009; Ye et al., 2012). On the other hand, genes related to the cell wall and sugar and nitrogen metabolism were upregulated (Hoeberichts et al., 2017). This is in line with previous studies explaining that cell wall rearrangements for permeability to water and other small molecules in cell-to-cell communication are necessary for endodormancy release (van der Schoot and Rinne, 2011). In the kiwi study, when Erger^®^ was applied, more than 30 MADS-box sequences were found in the transcriptome assembly (Hoeberichts et al., 2017). These transcription factors have been widely described as being implicated in the regulation of endodormancy and flowering time in various species, such as peach, almond and kiwi (Li et al., 2009; Varkonyi-Gasic et al., 2011; Prudencio et al., 2020). In addition, in red-rice seeds, processes to obtain energy, like glycolysis, were upregulated during endodormancy release (Gianinetti et al., 2018). This also agrees with other rearrangements in carbohydrate metabolism that have been observed in some perennial crops like grapevine and almond (Davies and Böttcher, 2009; Guillamón et al., 2020).

## Conclusion

Production in temperate fruit trees like *Prunus* species depends on successful flowering, which can only occur after successful endodormancy release. In recent years, global warming has endangered this process, producing problems in flowering, and production. To palliate this issue, different agrochemicals have been released. Nevertheless, the most effective agrochemical released to date, hydrogen cyanamide, has proven to be toxic. New environmentally friendly agrochemicals have therefore been released as alternatives to hydrogen cyanamide. Among them, Erger^®^ has shown the most effectiveness in advancing endodormancy release. Moreover, recent advances in transcriptomics and metabolomics have indicated certain genes and metabolites that are key factors in endodormancy release. These recent discoveries may be the first step in developing new environmentally friendly agrochemicals focused on the variations observed in metabolism during endodormancy release. Finally, it must be said that more assays with more species, cultivars and agrochemicals in different weather conditions should be performed in order to obtain more valuable and useful data with the aim of releasing new efficient green agrochemicals.

## Author Contributions

JG, FD, and RS-P wrote the manuscript. RS-P agreed to serve as the author responsible for contact and ensures communication. All authors contributed to the article and approved the submitted version.

## Conflict of Interest

The authors declare that the research was conducted in the absence of any commercial or financial relationships that could be construed as a potential conflict of interest.

## Publisher’s Note

All claims expressed in this article are solely those of the authors and do not necessarily represent those of their affiliated organizations, or those of the publisher, the editors and the reviewers. Any product that may be evaluated in this article, or claim that may be made by its manufacturer, is not guaranteed or endorsed by the publisher.
